# Supplementary material for: Safety of Tacrolimus Monotherapy within 12 Months after Liver Transplantation in the Era of Reduced Tacrolimus and Mycophenolate Mofetil: National Registry Study
Source: J Clin Med. 2022 May 17;11(10):2806. doi: 10.3390/jcm11102806 (PMC9145025; doi:10.3390/jcm11102806)
Supplement: Supplementary file 1 [file jcm-11-02806-s001.zip › jcm-1701230-supplementary.pdf]

Figure S1. Detailed number of patients along the process of matched analysis on time-conditional propensity score

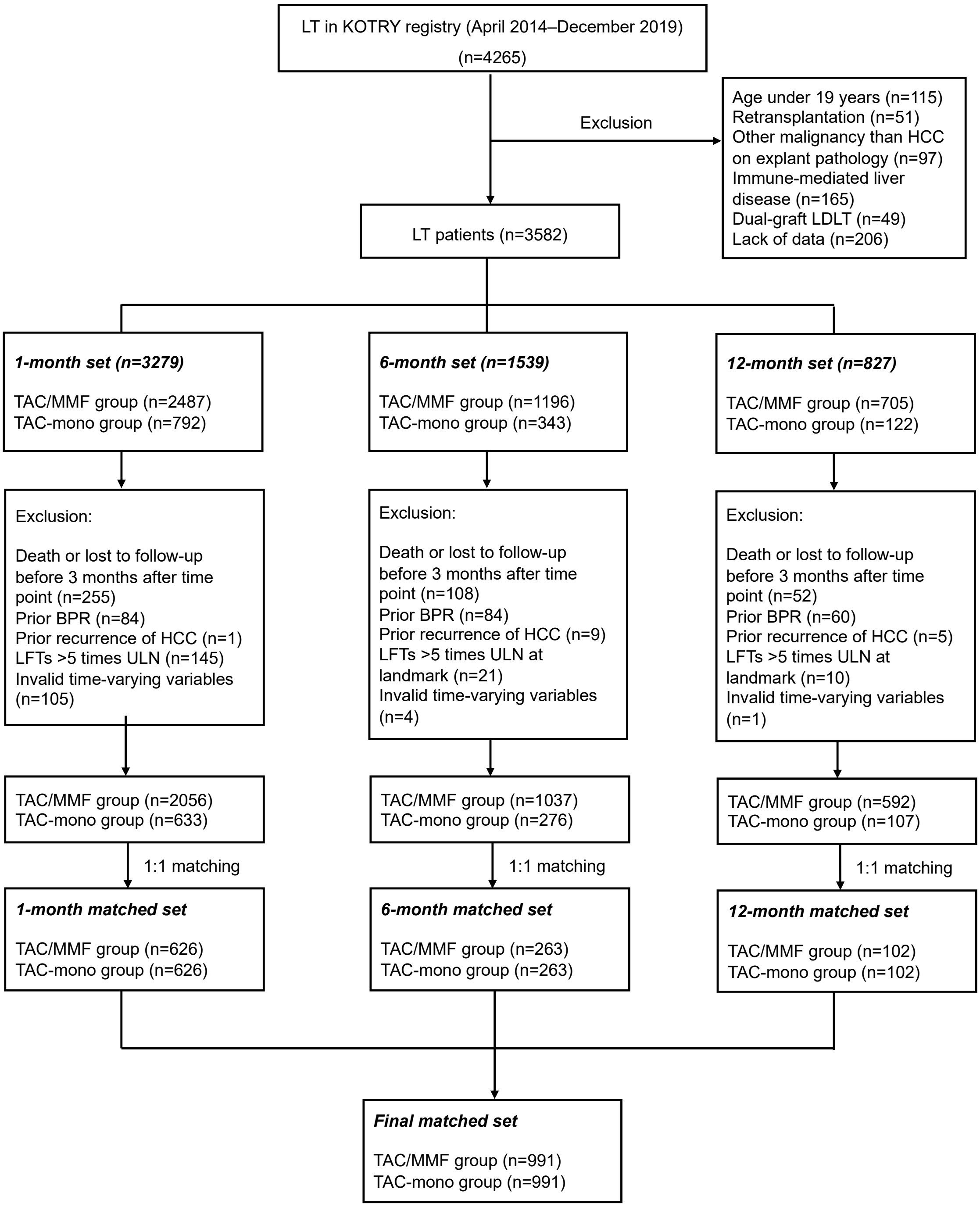

TAC/MMF users who were selected as matched comparators for given TAC monotherapy users once were not considered as possible comparators in subsequent

matching process. Matched TAC/MMF users who changed to TAC monotherapy during follow up were censored at the time of switch and included as TAC monotherapy group in the next matching set. TAC monotherapy users were matched to TAC/MMF users at 1:1 ratio with time-conditional propensity scores which was generated with baseline- and time-varying variables measured at each time points, including Karnofsky performance status score, glucocorticoid use, liver function tests, eGFR, and infection before matched time points. During the matching process, patients outside of balance for matching variables were discarded in both groups

BPR, biopsy-proven rejection; IS, immunosuppression; KOTRY, Korean Organ Transplantation Registry; LDLT, living donor liver transplantation; LFT, liver function test; LT, liver transplantation; MMF, mycophenolate mofetil; TAC, tacrolimus; ULN, upper limit of normal.

Figure S2. Balance of covariates used for calculating time-conditional propensity scores after matching.

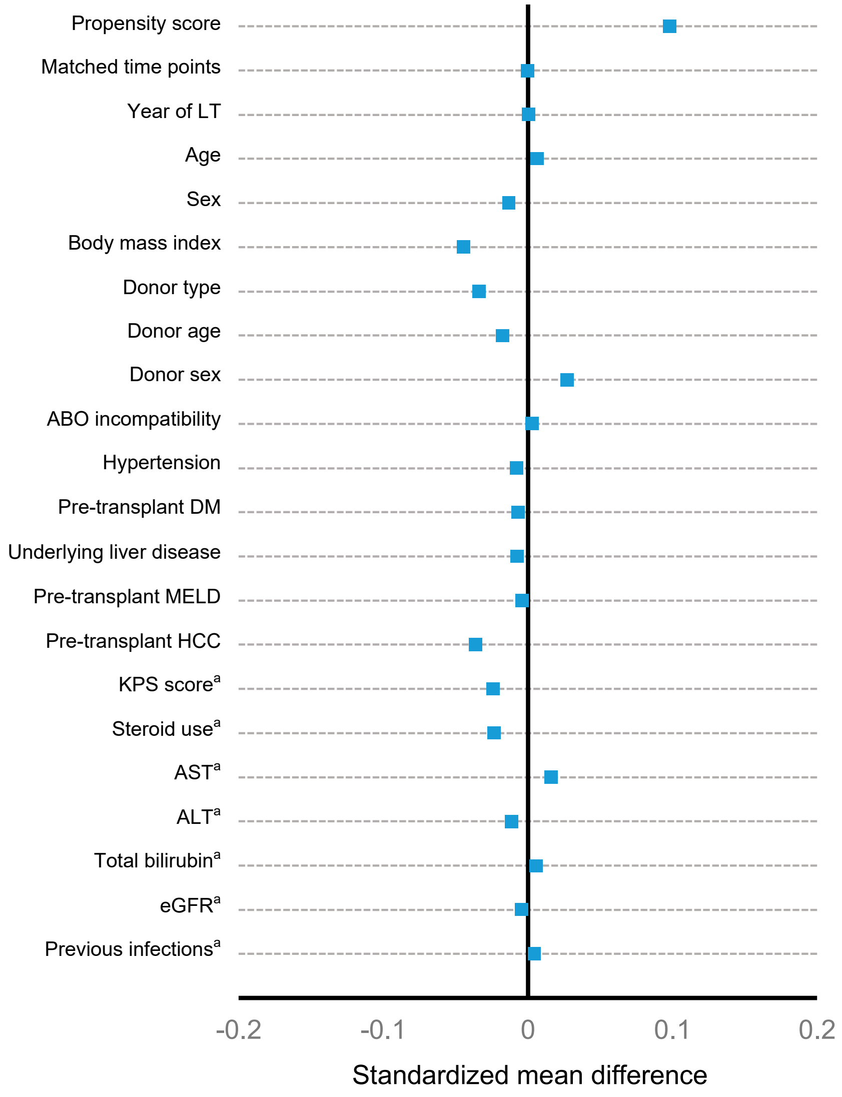

Standardized mean difference values between -0.1 and 0.1 were considered adequate matching.

[a] Time-varying variables at matched time points.

ALT, alanine aminotransferase; AST, aspartate aminotransferase; DM, diabetes mellitus; eGFR, estimated glomerular filtration rate; HCC, hepatocellular carcinoma;

LT, liver transplantation; MELD, Model for End-stage Liver Disease; MMF, mycophenolate mofetil; TAC, tacrolimus.

Figure S3. Distribution of immunosuppressive regimens in 3434 liver transplant recipients in the Korean Organ Transplantation Registry between April 2014 and December 2018.

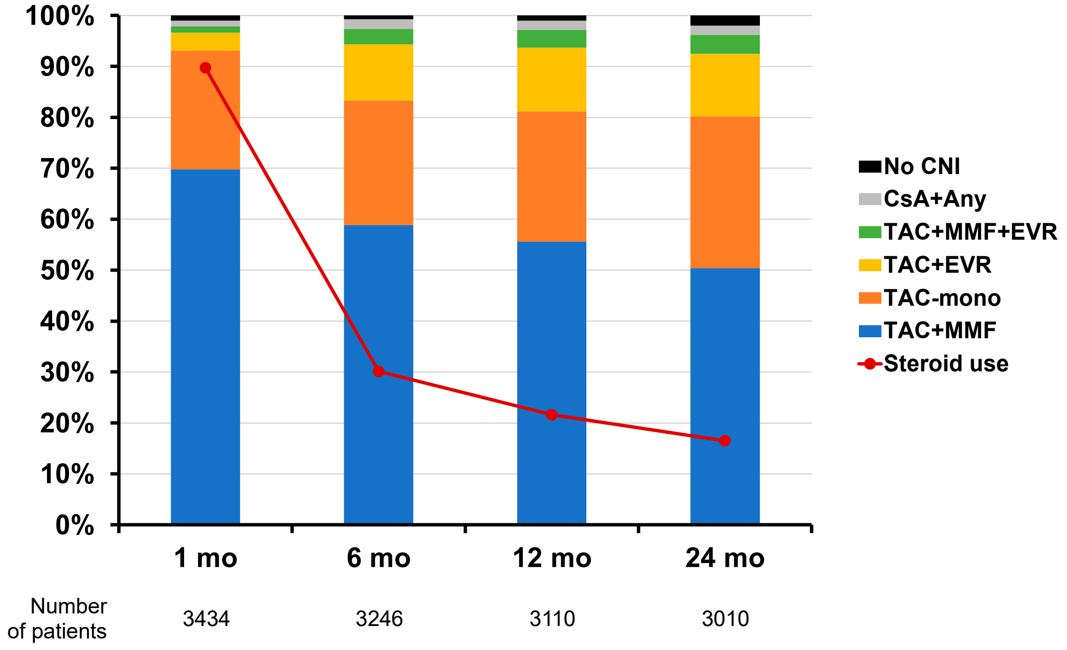

CNI, calcineurin inhibitor; CsA, cyclosporine A; EVR, everolimus; MMF, mycophenolate mofetil; TAC, tacrolimus.
